# Mimicking Natural Microenvironments: Design of 3D-Aligned Hybrid Scaffold for Dentin Regeneration

**DOI:** 10.3389/fbioe.2020.00836

**Published:** 2020-07-23

**Authors:** Elisabetta Campodoni, Samuele M. Dozio, Silvia Panseri, Monica Montesi, Anna Tampieri, Monica Sandri

**Affiliations:** Institute of Science and Technology for Ceramics, National Research Council, ISTEC-CNR, Faenza, Italy

**Keywords:** dentin regeneration, biomimetic scaffold, cross-linking, aligned structure, bio-inspired process

## Abstract

Tooth loss is a common consequence of a huge number of causes and can decrease the quality of humans’ life. Tooth is a complex organ composed of soft connective tissues and mineralized tissues of which dentin is the most voluminous component whose formation is regulated by a very complex process displaying several similarities with osteogenesis. Calcium phosphates, in particular hydroxyapatite (HA), is the phase present in higher amount into the structure of dentin, characterized by microscopic longitudinal dentinal tubules. To address the challenge of dental tissue regeneration, here we propose a novel biomimetic approach, to design hybrid scaffolds resembling the physico-chemical features of the natural mineralized tissues, suitable to recreate an appropriate microenvironment that stimulates cell colonization and proliferation, therefore effective for improving regenerative approach in dental applications. Biomineralization is the adopted synthesis as a nature inspired process consisting in the nucleation of magnesium-doped-hydroxyapatite (MgHA) nanocrystals on the gelatin (Gel) matrix generating hybrid flakes (Gel/MgHA) featured by a Gel:MgHA weight ratio close to 20:80 and size of 50–70 μm. Chemical and topotactic constrains affect the formation of MgHA mineral phase on the organic template, generating quasi-amorphous MgHA as revealed by XRD analysis and Ca/P ratio lower than 1.67, resembling the chemical and biological features of the natural apatite. The Gel/MgHA was then merged into the polymeric blend made of chitosan (Chit) and Gel to obtain a 3D porous scaffold with polymers: MgHA weight ratio of 40:60 and featured by an aligned porous structure as obtained by controlled freeze-drying process. The overall composite shows a swelling ratio of about 15 times after 6 h in PBS. The chemical stability was assured by means of a dehydrothermal cross-linking treatment (DHT) keeping the degradation lower than 20% after 28 days, while cell adhesion and proliferation were evaluated using a mouse fibroblast cell line.

## Introduction

Dentin, a calcified tissue underlying to the enamel, forms the bulk of the tooth and has a composition very similar to bone, in fact, it is mainly composed of a collagen matrix and nanocrystals of hydroxyapatite (HA: ∼75 wt% mineral, 20 wt% organic, 5 wt% water) (Boskey, 2007; Haumschild and Haumschild, 2009). Compared to bone, the apatite of the dentin has the same crystallographic orientation, but it is present in higher amount, in fact, it is nucleated not only onto the collagen fibrils but also in an additional space between them and this is responsible for its higher toughness (Burwell et al., 2012). High toughness together with the crack-arrest characteristic of the region between enamel and dentin, called dentin-enamel junction (DEJ), confer to the tooth a good fracture resistance (Wegst et al., 2015). Together with good mechanical properties the dentin shows also a specific structure arranged in channels called dentinal tubules (Wegst et al., 2015) that are formed during the biomineralization process, consisting in a complex cascade of phenomena generating nano-structured hybrids hierarchically organized from the nano to the macro-scale (Weiner, 2008; Sprio et al., 2016). Partial or full edentulism is a common result of several pathological conditions such as periodontal disease, profound caries, trauma but it is also due to a variety of physiological causes that decrease the individuals’ quality of life. The current solutions are still based on fixed prosthesis and full/partial dentures; however, due to high limitation of these solution, in recent research work the use of biomaterials in form of scaffold, stem cells and signaling molecules, are emerging (Hakki et al., 2016). Biomaterials are used to provide an appropriate support and microenvironment for cells to guide the regeneration of a well-structured organ in term of chemistry, mechanical strength and suitable 3D architecture (Eslahi et al., 2016). Among biomaterials, Ca-P ceramic based scaffolds present several advantages such as bioactivity, tunable porous structure for cell colonization and ability to support osteo/odontogenic differentiation (Bakopoulou et al., 2016).

Biomineralization process, mimicking the process of the dentin formation, is proposed, i.e., the nucleation of hydroxyapatitic phase onto organic templates, generating hybrid biomaterials with excellent biomimicry in term of chemistry and bio-resorbability (Olszta et al., 2007; Weiner, 2008; Sprio et al., 2016). The mineral phase synthetized through this process is very close to the biological apatite and it widely differs from the synthetic stoichiometric HA [Ca_10_(PO_4_)(OH)_2_] (Iafisco et al., 2014). Indeed it is characterized by nano-size crystals with low level of structural order and also several ions (e.g., CO${}_{3}^{2-}$, Na^+^, K^+^, and Mg^2+^) present in the physiological environment partially substituting Ca^2+^, PO${}_{4}^{3-}$, and OH^–^ ions (Landi et al., 2006; Tampieri et al., 2008; Panseri et al., 2012; Gómez-Morales et al., 2013; Ramírez-Rodríguez et al., 2016). Such apatitic phase, called biomimetic apatite, exhibit high solubility and the ability to deliver specific biological signals to cells, which are characteristics not found in synthetic analogs (Sprio et al., 2016).

Here, we propose a novel biomimetic approach to design hybrid biomaterials able to recreate a microenvironment delivering specific biological signals to cells, therefore effective for stimulating cell colonization and proliferation, and for improving regenerative approach in dental applications.

With the aim to develop scaffolds reproducing a proper microenvironment for cells also in terms of morphology and microstructure, thus ideal for guiding the whole process of dentin regeneration, all the following “key” requirements were considered: (i) the development of a well aligned porous morphology mimicking the typical dentin tubules; (ii) the achievement of hybrid materials with high content of bioactive mineral phase, mimicking the composition of dentin; and (iii) the reaching of suitable stability in physiological condition and good mechanical performance (Liu et al., 2016).

Biomineralization process was applied on gelatin (Gel), selected as the main biopolymeric template: the resulting hybrid mineralized flakes, were made of Gel and Mg-doped-hydroxyapatite nanocrystals (Gel/MgHA, 20:80 weight ratio). Their hybrid nature and high capability to adsorb fluids, allow to obtain a stable suspension by dispersing them in a polymeric blend made of gelatin and chitosan (Gel-Chit). Finally, a controlled freeze-drying process is the key step to shape a 3D homogeneous and porous device with the right morphology, stability and mechanical performances.

Among biopolymers, gelatin (Gel) and chitosan (Chit) were chosen because Gel has a lot of similarity with collagen, it is obtained by collagen denaturation, but maintaining some important properties such as biocompatibility, biodegradability and the ability to be mineralized (Hoque et al., 2015). However, since Gel has poor mechanical properties and stability (Boanini et al., 2010), Chit has been associated to reinforce the blend. Chit is a natural polysaccharide composed by glucosamine and N-acetylglucosamine, obtained by deacetylation of chitin (Zhao et al., 2002); it is synthesized by an enormous number of living organisms with the main function of reinforcing element. Both polymers are suitable to customize complex 3D porous scaffolds (Huang et al., 2005; Bi et al., 2011; Davidenko et al., 2015; Prata and Grosso, 2015; Krishnakumar et al., 2018; Campodoni et al., 2019) with interconnected and spatially organized by means of directional freezing and sublimation step during the freeze-drying process, and to be stabilized (Landi et al., 2008; Bi et al., 2011; Krishnakumar et al., 2018; Campodoni et al., 2019) by a dehydrothermal (DHT) cross-linking treatment. Finally, scaffold was chemically, morphologically and mechanically studied and a versatile and reliable mouse fibroblast cell-line was selected to provide a first insight on cytotoxicity and the nature of cell-material interaction.

## Materials and Methods

### Materials

Type A pig skin gelatin (Gel) in powder form (mesh 4, bloom 280) was purchased from Italgelatine (Cuneo, Italy) and used to prepare an aqueous solution (12 wt%) at 40°C. Low molecular weight chitosan (Chit) in powder form (50,000–190,000 Da, deacetylation degree >75%) was supplied by Sigma-Aldrich (MO, United States) and used to prepare an aqueous acid solution (2 wt%) dissolving Chit into acetic acid solution (1 wt%). Common high-purity chemical reagents were purchased from Sigma-Aldrich. Ultrapure water (0.22 mS, 25°C) was used for all experiments.

### Development of Mineralized Gel/MgHA Hybrid Flakes

Hybrid compound made of MgHA nanocrystals grown on Gel matrix were obtained through biomineralization process. In detail, the heterogeneous nucleation of MgHA nanocrystals on assembling Gel matrix was achieved by means of neutralization reaction performed as follows. An aqueous acid solution was prepared mixing H_3_PO_4_ (2.31 g in 100 mL) in Gel aqueous solution (0.84 g in 33.5 mL) at room temperature meanwhile, a basic solution was prepared adding 0.34 g of MgCl_2_ to aqueous suspension of Ca(OH)_2_ (2.60 g in 167 mL) kept at room temperature (all reagents were supplied form Sigma-Aldrich, MO, United States). The acid solution was immediately dropped into the basic solution under constant hand stirring at room temperature. The composite was ripened in the mother liquor without stirring for 2 h, then collected by centrifugation, washed three times with distilled water and freeze-dried. Finally, a DHT treatment was carried out after freeze-drying to improve composite stability introducing the composites into an oven at 160°C for 48 h under vacuum (0.01 mbar).

### Development of Gel/MgHA@Gel-Chit 3D Composite

10 mL of Gel aqueous solution (12 wt%) were kept to 40°C before adding 20 mL of Chit solution (chit was dissolved in an 1% acetic water solution to obtain a solid concentration of 2 wt%) and subsequently, the blend (Gel-Chit) was mechanically mixed for 30 min. Simultaneously, an aqueous suspension of Gel/MgHA hybrid flakes (3.2 g in 66 mL) was prepared and homogenized by sonication (tip sonicator ultrasonic processor VCX130, Sonics & Materials, United States) in ice bath for 10 min to disperse and disaggregate the Gel/MgHA clusters. Gel-Chit blend was added to Gel/MgHA under magnetic stirring at 37°C overnight to homogenize and blend the final hybrid composite (Gel/MgHA@Gel-Chit). The 3D structure was achieved by means of freeze-drying after introducing the composite into Teflon molds. The freeze-drying cycle was performed with a controlled freezing ramp (50°C/h) until −40°C and with a controlled heating ramp of 5°C/h from −40 to −5°C and 3°C/h until 20°C for about 3 days under vacuum conditions (*P* = 0.086 mbar). Finally, Gel/MgHA@Gel-Chit 3D composite underwent DHT treatment (160°C for 48 h under a pressure of 0.01 mbar) for the improving of scaffold stability thanks to covalent bonds formation within polymeric components.

### Development of Gel-Chit 3D Scaffold as Control

10 mL of Gel solution (12 wt%) were kept to 40°C before adding 20 mL of Chit solution (chit was dissolved in an 1% acetic water solution to obtain a solid concentration of 2 wt%) and subsequently, the blend (Gel-Chit) was mechanically mixed for 30 min. The blend was kept under magnetic stirring at 37°C overnight to homogenize and blend the final composite (Gel-Chit). The 3D structure was realized by means of freeze-drying after introducing the composite into Teflon molds. The freeze-drying cycle is performed with a controlled freezing ramp (50°C/h) until −40°C and with a controlled heating ramp of 5°C/h from −40 to −5°C and 3°C/h until 20°C for about 3 days under vacuum conditions (*P* = 0.086 mbar). Finally, Gel-Chit 3D scaffold underwent DHT treatment (160°C for 48 h under a pressure of 0.01 mbar) for the improvement of scaffold stability thanks to covalent bonds formation.

### Scaffold Characterization

#### Chemical–Physical Characterization

The samples X-ray diffraction (XRD) patterns were recorded by using a D8 Advance diffractometer (Bruker, Karlsruhe, Germany) equipped with a Lynx-eye position-sensitive detector (Cu Kα radiation, λ = 1.54178 Å) generated at 40 kV and 40 mA. XRD spectra were recorded in the 2θ range from 20° to 80° with a step size (2θ) of 0.02° and a counting time of 0.5 s. The thermal properties of the samples were measured using STA 449/C Jupiter (Netzsch, Germany). Simultaneous thermal gravimetric analysis (TGA) and differential scanning calorimetry (DSC) were carried out in alumina crucibles from room temperature to 1200°C at a heating rate of 10°C/min in nitrogen flow. The weight of the sample was approximately 10 mg. Fourier transform infrared (FTIR) spectra of KBr disks were collected by using a Nicolet 380 spectrometer (Thermo Fisher Scientific Inc., Waltham, MA, United States) working in the range of wavenumbers 4000–400 cm^–1^ at a resolution of 4 cm^–1^. A finely ground, approximately 1 wt% mixture of the sample in KBr was pressed into a transparent disk using a hydraulic press and applying a pressure of 48.6 psi (670 MPa). A pure KBr disk was used as blank. Calcium, phosphate and magnesium contents were determined by inductively coupled plasma optical emission spectrometry (ICP-OES) using a Liberty 200 spectrometer (Varian, Palo Alto, United States). An aliquot of 20 mg of sample was dissolved in 50 mL of a 1 wt% HNO_3_ solution prior the analysis.

#### Morphological Evaluation

The scaffold morphology and the pore size were observed by Inverted Ti-E fluorescence microscope (Nikon) and by environmental scanning electron microscopy (ESEM) (Quanta 600 FEG, FEI Company, Hillsboro, OR, United States). In ESEM microscopy, the specimens were mounted on aluminum stubs using carbon tape, and they were covered with a coating of Au using coating units Polaron Sputter Coater E5100 (Polaron Equipment, Watford, Hertfordshire, United Kingdom). The porosity was evaluated by two different methods: the density method and the water squeezing method. The density method measures the scaffold density and estimates the porosity by weighting the dried scaffold and measuring its volume in order to evaluate its absolute density through the equation:

$$\rho =\frac{W}{\pi \times {\left(\frac{D}{2}\right)}^{2}\times H}$$

where *W* is the weight of the scaffold, *D* is the diameter and *H* is the height of the scaffold (Mao et al., 2003). The absolute density was then divided into the absolute theoretical density of the material that was evaluated with the different compositions and the different absolute theoretical density of the reagents. Finally, with the absolute density, it was possible to evaluate the porosity of the scaffold. The values were expressed as the mean ± standard error (*n* = 3).

The water squeezing method measures instead the amount of water inside a scaffold before and after scaffold squeezing. The method is based on the principle that in the scaffold water is present both in polymer bounds and in small and big pores. The water residing in the macropores can be used to quantify the porosity requirement for cells penetration and proliferation. To measure the macropores volume percentage the scaffold was equilibrated in deionized water for an hour and weighed (*M*_*swollen*_), then squeezed to remove water in pores and weighed again (*M*_*squeezed*_). Macropores volume was calculated using the following equation:

$$\mathrm{Macropore}\,\mathrm{volume}\,\mathrm{percentage}=\frac{\left(M_{\text{swollen}}-M_{\text{squeezed}}\right)}{M_{\text{swollen}}}\times 100$$

The values were expressed as the mean ± standard error (*n* = 3) (Arora et al., 2015).

#### Stability Evaluation

The chemically effective crosslink density of the scaffolds was evaluated by a chemical assay based on the decrease of free primary amines (−NH_2_) or free carboxylic groups (−COOH), resulting from crosslinking reactions. In this study, the concentration of free −NH_2_ in uncrosslinked and crosslinked scaffolds was measured using a 2, 4, 6-Trinitrobenzenesulfonic acid (TNBS) assay, according to a protocol reported in the literature. Briefly, to each 5 mg of sample, 1 mL of a 4% (w/v) NaHCO_3_ solution was added. After 30 min, 1 mL of a freshly prepared solution of 0.5% (w/v) TNBS was added. The reaction mixture was heated at 40°C for 2 h and then 3 mL of 6M HCl solution were added. The samples were hydrolyzed at 60°C for 90 min. The reaction mixture was diluted 1:1 with distilled water, cooled to room temperature and the absorbance at 415 nm was measured using an UV–visible spectrophotometer (Perkin-Elmer Lambda 35, Italy). Blank control samples were prepared with the same procedure without scaffolds. The absorbance of the blank samples was then subtracted from each sample absorbance. Each sample measurements were run in triplicates (Arora et al., 2015).

The cross-linking percentage (CD) was evaluated with the following equation:

$$CD\left(\%\right)=\left(1-\frac{\mathrm{Absorbance}\,\mathrm{of}\,\mathrm{cross}-\mathrm{linked}\,\mathrm{sample}\,s}{\mathrm{Absorbance}\,\mathrm{of}\,\mathrm{non}-\mathrm{cross}-\mathrm{linked}\,\mathrm{controls}}\right)\times 100$$

In swelling test, the 3D composites were immersed in phosphate buffered saline (PBS) (pH 7.2) in presence of 0.1% (w/v) NaN_3_ at 37°C. At specific time intervals, the samples were taken out from the swelling medium and were blotted with a piece of paper to remove surface droplets. The swelling ratio (Qs) was evaluated using the following equation:

$$Q\,s=\frac{W_{s}-W_{d}}{W_{d}}$$

where *W*_*s*_ was the weight of the swollen sample at a specific time point and *W*_*d*_ was the initial weight of the dried sample (Campodoni et al., 2019).

In degradation test the 3D composite were immersed in PBS (pH 7.2) in the presence of 0.1% (w/v) NaN_3_ at 37°C. At specific time intervals, the samples were taken out from the medium and were washed twice with milliQ water. The samples were freeze-dried for 2 days and then weighed. The degradation percentage (D) was evaluated using the following equation:

$$D\left(\%\right)=\frac{W_{i}-W_{f}}{W_{i}}\times 100$$

where *W*_*i*_ was the initial weight of the dried sample and *W*_*f*_ was the weight of the freezer-dried sample at a specific time point (Tampieri et al., 2014).

#### Mechanical Properties

The mechanical properties were evaluated by DMA Q800 dynamic mechanical analyzer (TA instruments, IT) in the compressive mode testing samples with a diameter of 7–8 mm and a height of 4–6 mm. The measurements were carried out at 37°C and the samples were tested wet after immersion overnight in PBS (Sigma-Aldrich) at 37°C. In all experiments, a small preload (0.005 N) was applied to each sample to ensure that the entire scaffold surface was in contact with the compression plates before testing. The Young modulus was evaluated by means of a stress–strain test carried out after an isothermal period of 5 min, with a force ramp rate of 0.5 N/min close to 8 N. The slope of the linear fit line was calculated in a strain range from 0 to 10% (*n* = 5) (Arora et al., 2015). The viscoelastic measurements were carried out by multi-frequency tests, the spectra were obtained during a frequency scan between 0.1 and 10 Hz, and the experiments were performed under a constant strain amplitude (75 um) (*n* = 5) (Yan et al., 2012). The creep tests were carried out at different stress in the range of 0.001–0.1 MPa to study the linearity zone and choose the same stress (0.01 MPa) to use in the following creep test. The samples, after an isothermal period at 37°C of 5 min, were subjected at the defined stress for 15 min and afterwards they were left without any stress for 15 min (recovery time) (*n* = 3) (Campodoni et al., 2019).

### Preliminary *in vitro* Study

#### Cell Culture and Scaffold Seeding

Mouse Fibroblasts (mBALB/3T3 clone A31, ATCC) were cultured in Dulbecco Modified Eagle Medium high glucose (DMEM, Gibco), containing penicillin–streptomycin (100 U/mL–100 μg/mL) supplemented with 10% calf bovine serum and maintained at 37°C in an atmosphere of 5% CO_2_ and controlled humidity. Cells were detached from culture flasks by trypsinization and centrifuged. Cell number and viability were assessed with trypan blue dye exclusion test. Gel/MgHA@Gel-Chit scaffolds (8.00 mm diameter and 4.00 mm high) were sterilized with γ-rays. Samples were placed one per well in a 24-well plate and pre-soaked in culture medium for 24 h. Each scaffold was seeded by dropping 20 μL of cell suspension (5.0 × 10^4^ cells) onto the upper surface, allowing cell attachment for 30 min, before addition into each well 1.5 mL of cell culture medium. The medium was changed every 3 days. All cell handling procedures were performed in a sterile laminar flow hood. All cell culture incubation steps were performed at 37°C with 5% CO_2_.

#### Cell Viability Assay

Quantitative cell viability was assessed using the MTT assay. The MTT reagent (3-(4,5-dimethylthiazol-2-yl)-2,5-diphenyltetrazolium bromide) was prepared at 5 mg/mL in 1× PBS, and added at 1:10 ratio per well and kept 2 h at 37°C. In this assay, the metabolically active cells react with the tetrazolium salt in the MTT reagent to produce formazan crystals. Then, scaffolds were transferred to a tube containing 1 mL of dimethyl sulfoxide (DMSO) that dissolved the crystals. Two hundred microliters of supernatant were transferred into a 96-well plate and the absorbance was read at 570 nm using a Multiskan FC Microplate Photometer (Thermo Scientific). The absorbance is directly proportional to the number of metabolically active cells. Six samples were analyzed after 1, 3 and 7 days. The results were expressed as mean values of absorbance ± standard error of the mean (SEM) plotted on a graph. Statistical analysis was performed by the GraphPad Prism software (version 6.0), with statistical significance set at *p* ≤ 0.005.

Qualitative cell viability was assessed using the Live/Dead assay kit (Invitrogen), performed according to the manufacturer instructions. Briefly, scaffolds were washed with 1× PBS for 5 min and incubated with calcein acetoxymethyl (calcein AM) 2 μM, green-fluorescent dye for living cells and ethidium homodimer-1 (EthD-1) 4 μM, red-fluorescent dye for dead cells, for 15 min at 37°C in the dark. Samples were rinsed in 1× PBS. The top surfaces of samples were examined as well as their centers by previous sagittal cut. Images were acquired by an inverted Ti-E fluorescence microscope (Nikon). One sample per time point (1, 3, 7) was analyzed.

#### Cell Morphology Analysis

Cell morphology was observed via ESEM analysis, the samples were washed with 0.1 M sodium cacodylate buffer pH 7.4 and fixed in 2.5% glutaraldehyde in 0.1 M sodium cacodylate buffer pH 7.4 for 2 h at 4°C, washed in 0.1 M sodium cacodylate buffer pH 7.4 and freeze-dried. Dehydrated samples were sputter coated with gold and observed using ESEM (ESEM Quanta 200, Fei).

## Results

### Development of Gel/MgHA Hybrid Flakes

**FIGURE 1:**
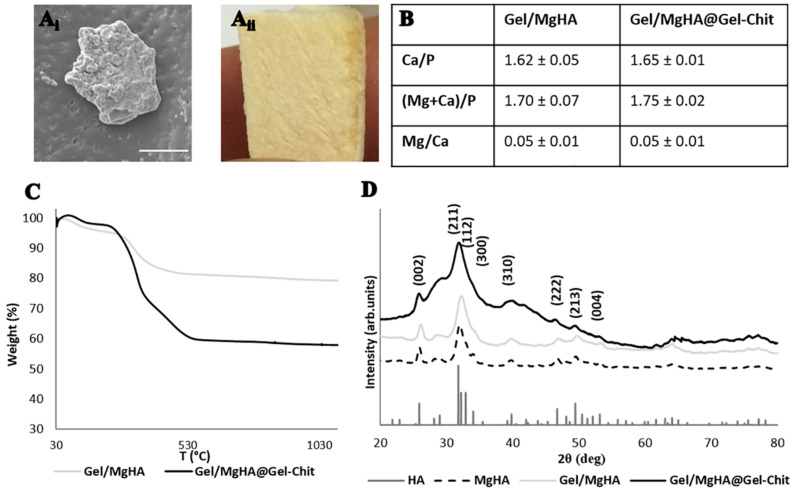
Results of analyses on Gel/MgHA hybrid flakes and Gel/MgHA@Gel-Chit 3D composite: **(Ai)** scanning electron micrographs of hybrid flakes (scale bar: 20 μm); **(Aii)** macroscopical image of 3D composite, sagittal section; **(B)** chemical composition obtained from ICP analysis; **(C)** thermal degradation curves obtained by TGA; **(D)** X-ray diffraction patterns of Gel/MgHA hybrid flakes and Gel/MgHA@Gel-Chit 3D composite compared to MgHA as control.

The biomineralization process generates high-mineralized hybrid flakes where apatite linked the major part of gelatin functional groups. ESEM image (Figure 1Ai) showed aggregates of mineral phase covering the Gel structure and creating flakes of about 50–70 μm. ICP analyses (Figure 1B) revealed the presence of Mg^2+^ ions (Mg/Ca = 0.05 mol ratio) and a Ca/P molar ratio of 1.62, lower than the stoichiometric HA molar ratio (Ca/P = 1.67) that confirm the presence of Mg^2+^ ions inside the lattice of HA and in substitution of Ca^2+^ ions. The interaction of apatite crystals with gelatin surface prevent their complete crystallization favoring the entrance of foreign ions and especially of Mg^2+^ that partially replaces Ca^2+^ creating hybrid composites Gel/MgHA whose chemistry and microstructural features are very similar to ones of young bones. XRD analyses, showed the typical diffraction pattern of a low crystalline apatite with a broadened profile (Figure 1D) (according to the PDF cards #09-0432) coherently with ICP data where a Ca/P molar ratio of 1.62 is typical of low crystalline hydroxyapatite partially substituted with Mg^2+^.

By comparing diffractograms of Gel/MgHA hybrid flakes and MgHA powder, prepared by biomineralization in the same synthesis conditions, the crystallinity extent is clearly lower in the hybrid due to the confinement exerted by the gelatin on the growing apatite nanocrystals. This explain the low level crystallization of the mineral phase and its high bioavailability.

TGA analysis of the hybrid flakes revealed high content of MgHA, close to 80 wt% (Figure 1C), essential to maintain an adequate amount of mineral phase in the final 3D scaffold obtained after mixing with the Gel-Chit blend.

### Development of Gel/MgHA@Gel-Chit 3D Composite

#### Morphological Evaluation

**FIGURE 2:**
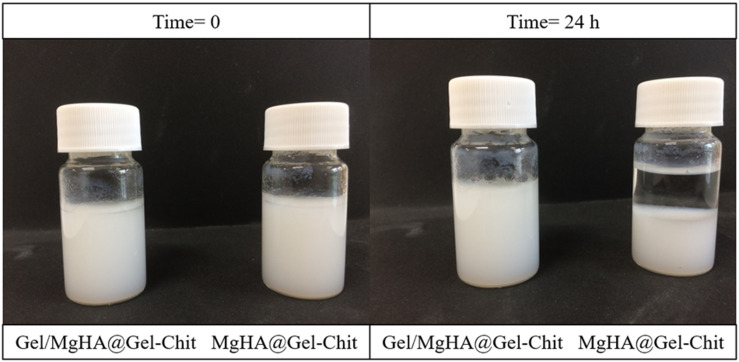
Macroscopical view of the comparison between MgHA and Gel/MgHA as mineral phase, observing the possible precipitation after 24 h.

As clearly shown in Figure 2, after 24 h the MgHA particles mixed in the Gel-Chit blend (MgHA@Gel-Chit) precipitates, while Gel/MgHA hybrid flakes mixed in the Gel-Chit blend (Gel/MgHA@Gel-Chit) are still completely suspended preserving a homogenous hydrogel. This is a very important aspect for the attainment of a homogenous and stable 3D structure by the subsequent freeze-drying step.

**FIGURE 3:**
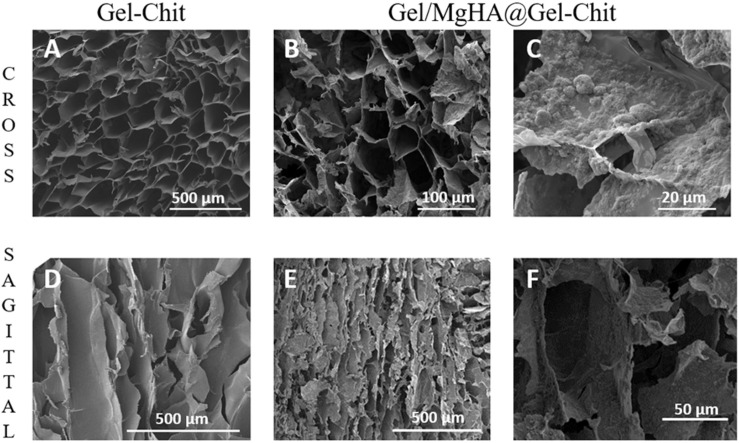
Scanning electron micrographs of different sections (cross and sagittal) of Gel-Chit as control scaffold **(A,D)** and Gel/MgHA@Gel-Chit composite **(B,C,E,F)**. Different magnifications are reported in the panel to better appreciate the whole morphology of Gel-Chit **(A,D)** and Gel/MgHA@Gel-Chit **(B,E)**. In detail, **(C,F)** the surface roughness and the mineral phase distribution of Gel/MgHA@Gel-Chit are reported.

Morphological evaluations on both polymeric (Gel-Chit) and composite (Gel/MgHA@Gel-Chit) highlight 3D highly aligned and homogenous structures characterized from well interconnected porosity, which is essential parameter for the scaffold colonization favoring dentin regeneration (Figure 3).

The good interaction between blend and hybrid flakes leads to homogeneous distribution of the mineral phase in the whole scaffold as demonstrated from the ESEM analysis of transversal slices (data no shown) and highlighted by ESEM images 3B and 3E.

The presence of the mineral phase is clearly visible on the surface of the pores (Figures 3C,F). Furthermore, despite of the presence of Gel/MgHA flakes the pores alignment is still clearly visible as Figure 3E shows and the presence of the mineral phase lead to a decrease in the pore size from about 132–62 μm (Figures 3A,B).

ESEM analyses revealed highly porous structures, confirmed also from the porosities measured with both density and water squeezing methods (Table 1). Both types of scaffolds have a high total porosity, close to 95%, given by nano and micro pores and evaluated by density method. The macro porosity, referred only to the pores effectively available for cell adhesion and migration inside the scaffold, was evaluated by water squeezing method. It was slightly lower but it maintained high values, around 80%. For both methods, Gel/MgHA@Gel-Chit scaffold highlighted higher porosity values in respect to the control Gel-Chit (Table 1).

**TABLE 1:** Dimensional properties (total porosity and macroporosity, density and pore size), swelling ratio and degradation percentage of Gel-Chit control scaffold and Gel/MgHA@Gel-Chit 3D composite (mean ± SD).

		**Gel-Chit**	**Gel/MgHA@Gel-Chit**
% Porosity	Density method (total porosity)	95.4 ± 0.1	97.6 ± 0.1
% Porosity	Water squeezing method	82.1 ± 3.3	87.2 ± 1.8
	Density (g/cm^3^)	4.59 ± 0.07	2.46 ± 0.16
	Pore size (μm)	132.08 ± 39.98	61.78 ± 15.14
	Swelling ratio at 6 h	12.2 ± 1.1	15.2 ± 1.3
	Degradation (%)	24.3 ± 1.7	16.9 ± 1.2

#### Chemical–Physical Evaluation

Although the integration of Gel/MgHA into blend to obtain the final 3D composite (Gel/MgHA@Gel-Chit), the mineral phase does not change its properties as ICP revealed (Figure 1C). The final product maintained a Ca/P ratio of 1.65 typical of low crystalline hydroxyapatite partially substituted with Mg^2+^. The presence of polymeric blend (Gel-Chit) decreased the ratio between polymer and mineral phase as confirmed from TGA that revealed an initial wt% ratio of 20/80 for Gel/MgHA hybrid flakes, which becomes 40/60 in the final device (Gel/MgHA@Gel-Chit), still adequate for hard tissue regeneration (Figure 1C). Analyzing the TGA profiles, different weight losses are clearly visible: they are ascribable to the breakage of inter- and intra-molecular hydrogen bonds of polymers and the release of water molecules (up to 200°C), to the degradation of Gel and Chit (around 300°C) and, at higher temperatures, to the pyrolysis of carbon residues (around 400°C).

**FIGURE 4:**
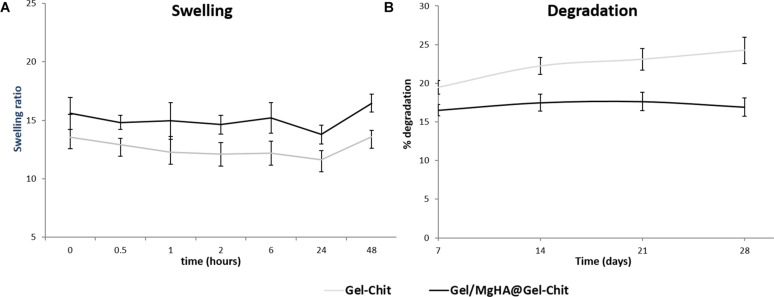
**(A)** Swelling ratio up to 48 h and **(B)** degradation profile up to 28 days of Gel/MgHA@Gel-Chit 3D composite obtained by biomineralization and blending processes compared to Gel-Chit as control scaffold (*n* = 3).

Furthermore, XRD analysis (Figure 1E), performed after mixing procedure with the blend Gel-Chit, confirmed the preservation of a low crystalline MgHA reporting the typical pattern characterized from wide peaks. For good performances in vivo, 3D scaffolds should show high swelling ability and low degradation rate thus allowing cell adhesion and proliferation. A good swelling can guarantee a superficial hydration layer favoring cell adhesion on the scaffold surface and a low degradation rate permits the cell proliferation, differentiation and new tissue formation before losing the 3D scaffold structure and support. From the analyses (Figure 4A), Gel/MgHA@Gel-Chit and the control Gel-Chit displayed a good swelling because both scaffolds absorb the medium about 12–15 times of their weight. In particular, both scaffold Gel-Chit and Gel/MgHA@Gel-Chit displayed a swelling ratio of 12.2 ± 1.1 and 15.2 ± 1.3, respectively after 6 h. Furthermore, both scaffolds degraded less than 20 wt% in 28 days, they demonstrate a degradation of 24.3 ± 1.7 and 16.9 ± 1.7%, respectively (Figure 4B). The presence of Gel/MgHA hybrid flakes influences both these properties: from one side, the hydrophilicity is increased and swelling improved; on the other side, the 3D composite is stabilized against degradation.

#### Mechanical Evaluation

**FIGURE 5:**
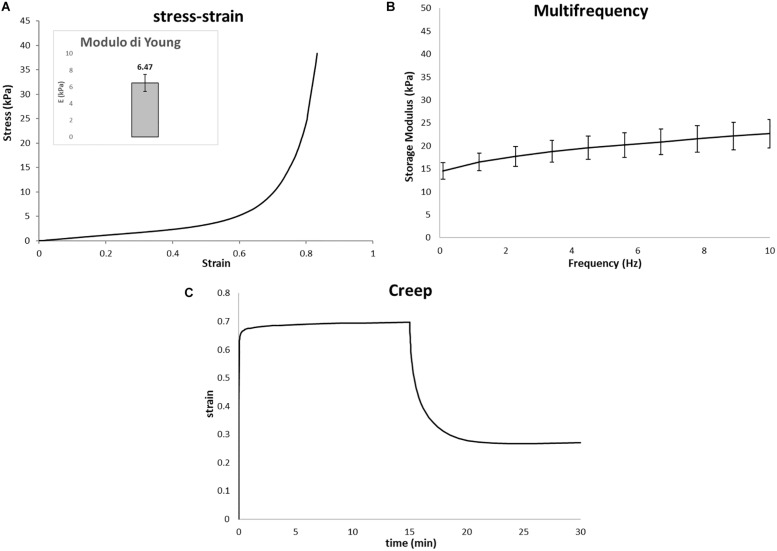
Representative mechanical properties of Gel/MgHA@Gel-Chit 3D composite performed after immersion in PBS at 37°C overnight using DMA instrument upon compressive mode. In detail: **(A)** average stress–strain curves, inset represent Young modulus calculated as the slope of the stress–strain curves in a range from 1 to 10%; **(B)** average storage modulus (E′) – frequency curves measured from 0.1 to 10 Hz; **(C)** average creep curves obtained after creep time of 15 min and recovery time of 15 min (*n* = 3, data are mean ± std).

The catastrophic failure plateau and the densification regions of the scaffolds can be observed from typical stress–strain curve as seen in Figure 5A. The Young’s modulus of the Gel/MgHA@Gel-Chit scaffolds, calculated between 1 and 15% strain value, is 6.12 kPa. The visco-elastic behavior of the scaffold is described by the storage modulus (E′) that increases with the increase of frequency (Figure 5B). In response to oscillating frequencies, Gel/MgHA@Gel-Chit shows significant variation in the E′ value: in the range 0.1–10 Hz, E′ value increases from 15.2 ± 3.6 to 24.1 ± 2.8 kPa. Finally, the creep tests (Figure 5C) show the behavior of the scaffold when it is subjected to a static stress for a specific time and the strain recovering after releasing of stress. Static stress was identified with a preliminary linearity study and corresponds to 0.005 MPa. During the compression of 15 min, Gel/MgHA@Gel-Chit reached a strain of 70%, however, after stress releasing, it recovers most of the original shape. The spectra show indeed that the final strain is about 28% and the strain recovery is close to 60%.

### *In vitro* Test of Gel/MgHA@Gel-Chit

**FIGURE 6:**
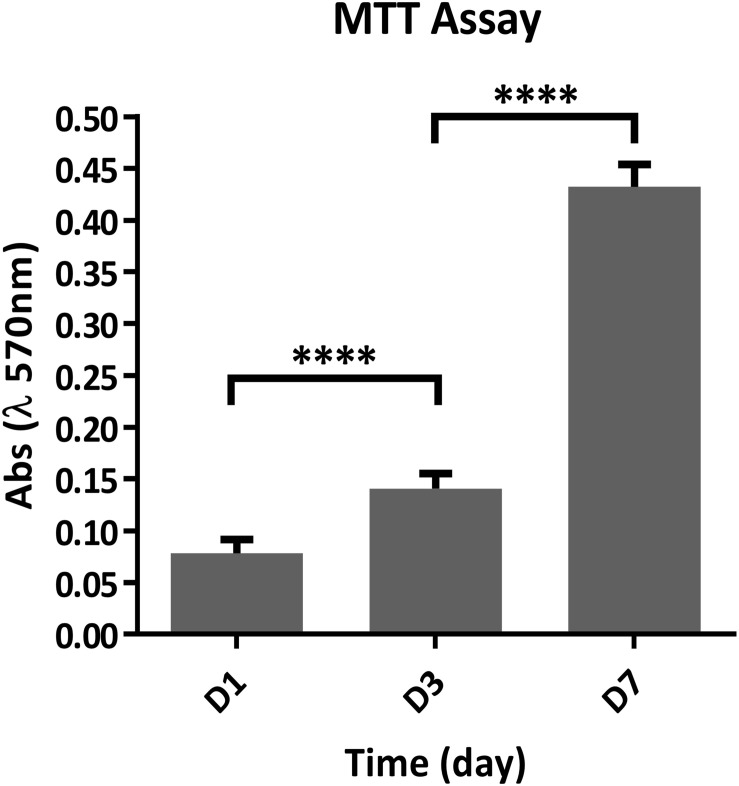
Quantitative analysis of cell proliferation using the MTT assay at 1, 3, and 7 days after seeding (*n* = 6). ****p ≤ 0.0001.

To assess whether the chemical composition of the biomaterials could lead to cytotoxicity, compromising the fibroblast cells (mBALB/3T3) viability, two types of assay have been performed. The quantitative cell viability and proliferation, assessed by MTT assay, showed a statistically significant increment over time of the number of live cells; indicating that the biomaterial does not compromise the cell viability and also significantly stimulate cells proliferation, with a *p*-value ≤ 0.0001 (Figure 6).

**FIGURE 7:**
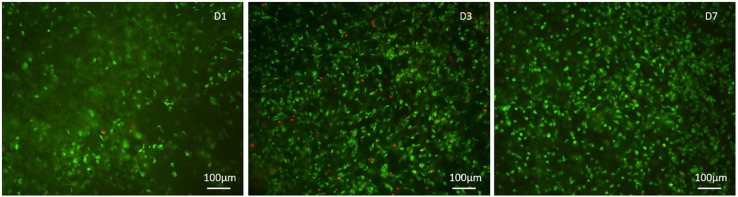
Qualitative cell viability analysis performed with the Live/Dead kit, calcein stains live cells in green, ethidium homodimer-1 stains dead cells in red at day 1, 3, and 7 after seeding.

The quantitative cell viability assay is supported by the qualitative assay performed using the Live/Dead assay kit, as it confirmed the presence of a greater number of live cells compared to the dead cells, increasing at every time point (Figure 7).

**FIGURE 8:**
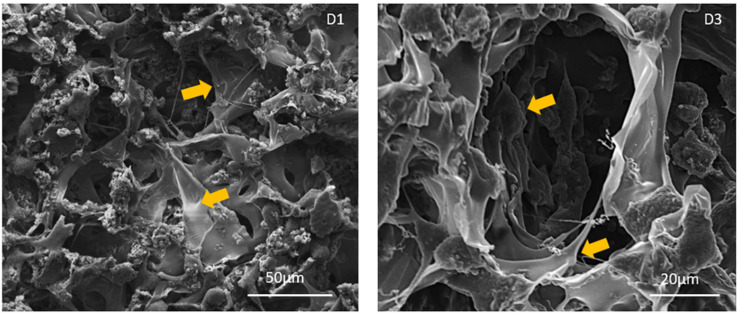
ESEM images of cell-seeded scaffolds at day 1 and 3 after seeding. Yellow arrows show cells stretched and anchored onto the scaffolds. No round shaped cells, lacking cytoplasmic extensions, were observed at each time point.

The morphology of fibroblast cells grown on the Gel/MgHA@Gel-Chit scaffold was evaluated via ESEM observations (Figure 8). The cells result well spreaded on the biomaterials showing a healthy and characteristic morphology. The evident and numerous cytoplasmic extensions observed can be considered an index of high cell/biomaterial interactions. It also revealed the absence of round-shaped cells, indicators of impaired anchoring capability due to lack of cell-material compatibility.

## Discussion

Starting from a careful study of the teeth morphology and composition, 3D composite with customized characteristics has been developed to reproduce the key features of the natural dentin, create an ideal microenvironment in terms of biochemical signals and microstructure and thus to have the requisites to participate to the complex regenerative cascade forming in vivo this tissue. More in details the aim of this work was to create a new biomaterial mimicking the dentin in terms of chemical composition and 3D architecture, recognizable by cells as a suitable microenvironment for adhesion, proliferation and differentiation (Palosaari et al., 2003). Following a biomimetic approach, the biomineralization process was applied to develop 3D composite (Gel/MgHA@Gel-Chit, 60/40 wt%) in which the features of the mineral and organic phases were selected to recreate the structure of dentin at different scale size (Roveri et al., 2003; Tampieri et al., 2003). In particular in order to provide 3D geometry able to resemble the physiological condition and to promote cells stemness maintenance (Farooque et al., 2014) Gel/MgHA (20/80 wt%) hybrid flakes had been blended into Gel-Chit matrix for the achievement of a stable and biomimetic 3D composite mimicking the composition and the structure of dentin. The reasons had led to choose Gel/MgHA hybrid flakes instead of MgHA particles as mineral phase were: (i) Gel, as template molecule for mineralization, improves the biomimetism of MgHA particles, allowing to obtain a mineral phase very close to the natural one in terms of crystallinity, chemical composition and morphology (Iafisco et al., 2014); and (ii) Gel/MgHA, because in form of hydrate flakes, allow to generate a more homogeneous and stable suspension due to the high affinity between the functional groups of Gel-Chit blend and mineralized Gel. The achievement of a stable suspension is crucial to obtain by freeze-drying a homogeneous and a well-ordered 3D-aligned porous scaffold. In particular, as inferable from the analyses reported in Figure 1, the mineral phase nucleated onto Gel was low crystalline MgHA, since the organic template constrained the nucleation and facilitated the entering of Mg^2+^ ions instead Ca^2+^ (5 molar %) inside the hydroxyapatite lattice.

On the other hand Gel was selected as a promising organic template for biomineralization process thanks to its similarity with collagen (Landi et al., 2008; Jiang et al., 2018; Lewis et al., 2018). However, gelatin shows specific different features compared to collagen: Gel (obtained by hydroxylation) when involved in the biomineralization procedure, has less inclination to assemble in 3D structures, but higher affinity to pin the mineral phase nucleation (Tampieri et al., 2011; Krishnakumar et al., 2017; Menale et al., 2018). As a consequence of this fact, differently from collagen (Roveri et al., 2003), the hybrid Gel/MgHA cannot be obtained as 3D assembled structure, but in form of mineralized flakes, containing much higher amount of mineral phase compared to collagen. Indeed, Gel exposes most of its functional groups to interact with calcium and phosphate ions and thus they are no longer available to induce the self-assembly process (Landi et al., 2008). To overcome the lacks of the 3D architecture, required to provide an appropriate micro-environment for cells growth, the strategy used in this work was to merge the highly mineralized hybrid flakes (Gel/MgHA) into a biopolymer matrix obtained by blending Gel and Chit (Gel-Chit) (Eslahi et al., 2016). The presence of Gel in Gel/MgHA hybrid flakes improves the swelling and favors the attainment of Gel/MgHA@Gel-Chit suspension characterized by high stability (Figure 2) leading to homogenous distribution of the mineral phase within the scaffold as SEM analysis confirmed (Figure 3E). The polymeric blend Gel-Chit was selected to obtain the 3D aligned micro-architecture resembling the dentinal microtubules. In fact Chit under acidic conditions has a positive charge that comes from the protonation of free amino groups and this allows the fabrication of homogeneous and stable composite when in contact with Gel, as consequence of the formation of hydrogen bonds within and between the polymers chains and also of electrostatic interactions between –COO^–^ of Gel and −NH3^+^ of Chit (Drury and Mooney, 2003; Kaczmarek et al., 2018). Those strong chemical interactions led to a spontaneous self-assembling of Gel and Chit molecules and the generation of a stable and homogeneous polymeric 3D composite (Zhao et al., 2002; Mohammadi et al., 2018).

The achievement of dry scaffolds with the desired 3D microarchitecture was realized by the freeze-drying technique where a directional freezing generated well-oriented and interconnected channel-like pores. The pores characteristics, i.e., size, homogeneity and orientation are dependent on ice crystal nucleation and growth. In particular, pore size is governed by freezing temperature, pore homogeneity is controlled by freezing rate, while pore orientation can be controlled by directional freezing which is in turn dependent by the conductivity of the mold containing the hydrogel during the freezing step. During the process, when the suspension was completely frozen, the temperature was increased until 15°C under vacuum to reach the triple point of water and remove the ice by sublimation. The ice crystals leave empty space in the composite matrix and produce porous interconnected structure that can be controlled and duly designed. Porosity is a key parameter in the regenerative task, because cells, by means of pores, can stick on the scaffold surface, grow and proliferate deep inside in the bulk (Mao et al., 2003; Landi et al., 2008; Bi et al., 2011; Kaczmarek et al., 2018). The homogeneity between the mineral phase and the polymer blend described above was confirmed by ESEM images (Figure 3), moreover, the presence of mineral phase and its interaction with the polymer blend did not interfere either with the formation of a 3D aligned porosity nor with the interconnection extent which in turn influenced the hydration capability of the scaffold (Yan et al., 2010, 2012). The DHT process, named zero-length cross-linking, was used to stabilize the scaffold against the enzymes attack and thus reduce the degradation rate. The process, promoting the formation of amides between the polymers’ functional groups (Li et al., 2017), during the DHT treatment, would have the effect of decreasing the hydrophilicity of the scaffold and consequently its swelling (Madaghiele et al., 2016; Shankar et al., 2017). To contrast this effect, we applied the DHT cross-linking on two different highly hydrophilic polymers very rich in functional groups. Consequently, the final scaffold showed high stability, thanks to the DHT process, whereas retaining a good level of swelling crucial to promote cells adhesion and tissue formation inside the 3D scaffold.

Finally, since Gel/MgHA@Gel-Chit scaffold is expected to be used in a hydrated environment and it starts to be reabsorbed during the formation of the new tissue, the evaluation of the biomechanical behavior in “*in vivo-like*” conditions was considered. The evaluation of Young’s modulus, viscoelastic properties and creep behavior indicated that the cross-linking technique and the mineral phase are two aspect influencing the mechanical behavior of this nanocomposite. Young’s modulus of 3D composite close to 6.47 kPa is lower than natural dentin, but it is adequate to bear a static stress as creep test demonstrated. Thus, after 15 min under a static stress (0.01 MPa), 3D composite is capable to recover most of the original shape since strain recovery is close to 60%. Both these properties (Young’s modulus and strain recovery) showed a material capable not only to withstand the compression, but also to maintain its shape due to the good elasticity in wet conditions. Translating these features in *in-vivo* condition, such a mechanical behavior of the scaffold can assure structural integrity and load-bearing properties to sustain cell colonization and proliferation during new tissue formation and scaffold degradation.

We demonstrated that the chemistry of Gel/MgHA@Gel-Chit 3D composite does not interfere with the cell viability, as proved by the preliminary results obtained in *the vitro* study. It is possible to assume that along with the chemical composition, both the structural topography and roughness of the scaffold contributed to the overall cell colonization, proliferation and interaction with the biomaterial as proved by the ESEM images showing good cells adhesion and healthy cytoplasmic spreading. Even if preliminary, the *in vitro* study demonstrated that this biomaterial exhibits promising features to assist not only cell interaction but also proliferation, giving particular relevance of this biomaterial for wide biomedical applications.

## Conclusion

A 3D composite with customized characteristics has been designed with the aim to develop a dentin-like scaffold for new approach in dental regenerative application. In particular, studying the teeth morphology and the dentin structure, a biomimetic approach was applied consisting in the nucleation of Mg-substituted HA on gelatin polymer, giving highly mineralized hybrid flakes (Gel/MgHA, 20/80 wt%) which are in turn embedded in a matrix formed of gelatin and chitosan blend, in order to develop the 3-D composite (Gel/MgHA@Gel-Chit, 40/60 wt%) with aligned porous microstructure.

Each component and process used to generate the final scaffold play a precise role:

(i) Gel/MgHA hybrid flakes are the result of biomineralization, where the mineral phase is confined at the nanosize level by the interaction with gelatin; such interaction, reducing the crystallinity, also promotes the substitution of Ca^2+^ with Mg^2+^ ions.
(ii) The mineral phase MgHA, resembling very well the mineral component of natural mineralized tissues, stabilizes the Gel/MgHA flakes against degradation and preserves the hydrophilic properties of the final composite.
(iii) The gelatin and chitosan, thanks to their interacting functional groups, form a 3D matrix (Gel-Chit) where the channel–like architecture can be formed by tuning the freeze-drying process.
(iv) The DHT process confers to the scaffold suitable degradation rate in compliance with tissue remodeling. In addition, it affects the elasticity, the shape maintenance and the resistance to compression making the scaffold able to sustain cell attachment and colonization in physiological environment.

Concluding, the Gel/MgHA@Gel-Chit 3D composite shows very good biocompatibility and can be considered a good candidate for dentin regeneration as well as for applications in the field of bone regeneration.

## Data Availability Statement

All datasets presented in this study are included in the article/supplementary material.

## Author Contributions

EC, MS, and AT conceived and designed the experiments and prepared the manuscript. SD, MM, and SP performed the *in vitro* cell culture experiments. All authors discussed the results.

## Conflict of Interest

The authors declare that the research was conducted in the absence of any commercial or financial relationships that could be construed as a potential conflict of interest.
